# Differential Regulation of Immune Signaling and Survival Response in *Drosophila melanogaster* Larvae upon *Steinernema carpocapsae* Nematode Infection

**DOI:** 10.3390/insects9010017

**Published:** 2018-02-08

**Authors:** Shruti Yadav, Sonali Gupta, Ioannis Eleftherianos

**Affiliations:** Insect Infection and Immunity Lab., Department of Biological Sciences, The George Washington University, Washington, DC 20052, USA; shrutiyadav@gwmail.gwu.edu (S.Y.); sonaligupta@gwmail.gwu.edu (S.G.)

**Keywords:** *Drosophila melanogaster*, *Steinernema carpocapsae*, immune signaling, immune response

## Abstract

*Drosophila melanogaster* is an excellent model to dissect the molecular components and pathways of the innate anti-pathogen immune response. The nematode parasite *Steinernema carpocapsae* and its mutualistic bacterium *Xenorhabdus nematophila* form a complex that is highly pathogenic to insects, including *D. melanogaster*. We have used symbiotic (carrying *X. nematophila*) and axenic (lacking *X. nematophila*) nematodes to probe the regulation of genes belonging to different immune signaling pathways in *D. melanogaster* larvae and assess the survival response of certain mutants to these pathogens. We found that both types of *S. carpocapsae* upregulate *MyD88* (Toll), but not *PGRP-LE* (Imd); whereas axenic *S. carpocapsae* strongly upregulate *Wengen* (Jnk), *Domeless* (Jak/Stat), *Dawdle* (TGFβ, Activin), and *Decapentaplegic* (TGFβ, BMP). We further found that inactivation of *Wengen* and *Decapentaplegic* confers a survival advantage to larvae infected with axenic *S. carpocapsae*, whereas mutating *PGRP-LE* promotes the survival of larvae infected with symbiotic nematodes.

## 1. Introduction

Entomopathogenic nematodes are natural parasites that infect a range of insect species [1,2,3]. In recent years, they have also emerged as excellent models to dissect the molecular basis of nematode parasitism [4,5]. The entomopathogenic nematode *Steinernema carpocapsae* forms an excellent tool to study the molecular interplay between insect hosts and nematode parasites [6,7]. *S. carpocapsae* nematodes have developed mutualistic relationship with the Gram-negative bacteria *Xenorhabdus nematophila*, which live in the gut of the parasite and are potent pathogens of insects [6,7,8,9]. The nematodes infect insects at the infective juvenile stage, which is analogous to the *Caenorhabditis dauer* stage [10]. Once the nematode gains entry into an insect, it expels its associated bacteria in the hemolymph and both the nematode and bacteria multiply and secrete virulence factors that disarm the insect’s immune system [4,9,11]. The bacteria also assist their nematode partner by producing molecules that break down insect tissues, thereby promoting feeding of the parasite and completion of its life cycle [12]. When the food source is depleted, the nematodes take up their mutualistic bacteria and exit the insect as infective juveniles in search of new susceptible hosts [10]. In addition, the development of tools to generate *S. carpocapsae* nematodes devoid of their mutualistic bacteria (axenic worms) has permitted elegant studies for the identification of host responses directed exclusively against the nematodes [13,14].

*Drosophila melanogaster* is a well-established insect model to investigate the genetic basis of the innate immune response to a wide range of pathogens, including parasitic nematodes [15]. Although the molecular players involved in the recognition and defense against nematodes are not fully characterized, recent studies have identified genes/gene families in *D. melanogaster* larvae and adult flies that are induced in response to nematode infections [7,16,17]. In recent years, the use of *S. carpocapsae* as model of nematode parasitism has led to the identification of potential anti-nematode factors in insect hosts [6,7,18]. A recent transcriptomic study has identified several *Drosophila* larval genes that could be involved in the recognition of and defense against *S. carpocapsae* nematodes [7]. 

Here, we exposed *D. melanogaster* larvae to *S. carpocapsae* symbiotic or axenic nematodes and analyzed the transcriptional regulation of *PGRP-LE* (Immune deficiency pathway, Imd), *MyD88* (Toll pathway), *Wengen* (*Wgn*, c-Jun N-terminal kinase signaling pathway; Jnk), *Domeless* (*Dome*, Janus kinase and signal transducer and activator of transcription pathway; Jak/Stat), *Dawdle* (*Daw*, Activin branch of the transforming growth factor beta pathway; TGFβ) and *Decapentaplegic* (*Dpp*, Bone Morphogenetic Protein branch; TGFβ pathway), which belong to different immune signaling pathways. We also tested the survival ability of *D. melanogaster* loss-of-function mutant larvae against *S. carpocapsae* to assess the participation of certain signaling pathways in the *D. melanogaster* response to this parasitic nematode.

## 2. Materials and Methods 

### 2.1. Fly Strains

*Drosophila melanogaster* strains used included Oregon, *w^1118^*, *yw*, *PGRP-LE* (Bloomington *Drosophila* Stock Center; 33055; FBgn0030695), *MyD88^c03881^* [19], *Wgn* (Vienna *Drosophila* Resource Center; v9152; FBgn0030941), *UAS-Dome^DN^* [20], *Daw* (Kyoto Stock Center; 113490; FBgn0031461), and *Dpp* (Bloomington *Drosophila* Stock Centre; 397; FBgn0000490) [21] (Table A1). *Wgn*-RNAi and UAS-*Dome^DN^* were crossed with ubiquitous Actin-Gal4 driver (y[1] w[*]; P{w[+mC] = Act5C-GAL4}25FO1/CyO, y[+]) [22]. Fly strains were grown on *Drosophila* media (Meidi LaboratoriesR Potomac, MD, USA) and approximately 10 granules of dry baker’s yeast. All fly stocks were maintained at 25 °C with a 12:12-h light:dark cycle. Late second-early third instar larvae were used for all experiments. 

### 2.2. Nematodes

The entomopathogenic nematodes *Steinernema carpocapsae* carrying the mutualistic bacteria *Xenorhabdus nematophila* (symbiotic worms) were reared in the larvae of the greater wax moth *Galleria mellonella* [23]. Axenic *S. carpocapsae* nematodes (free of *X. nematophila* bacteria) were cultured using a previously described protocol [14]. These nematodes were washed with 1% bleach, and then rinsed five times with water to remove any traces of bacteria or bleach. Infective juveniles between 2 and 3 weeks of age were used for all infection experiments. 

### 2.3. Gene Transcript Level Analysis

Reads Per Kilobase Million (RPKM) values for *PGRP-LE*, *Myd88*, *Wgn*, *Dome*, *Daw*, and *Dpp* genes were obtained from a recent RNA sequencing study [7], which involved the transcriptomic analysis of *D. melanogaster* larvae during the course of infection with 100 *S. carpocapsae* symbiotic or axenic nematodes. The RPKM values for genes from larvae that were infected with either symbiotic or axenic nematodes were calculated relative to the RPKM values of the uninfected controls, which were set to 1. 

### 2.4. Survival Experiments

Microtiter 96-well plates (Corning, Corning, NY, USA) were prepared by adding 100 µL of 1.25% agarose to each well. Ten *S. carpocapsae* symbiotic or axenic nematodes suspended in 10 µL of distilled water were added to each well containing a single larva. Addition of 10 µL of distilled water served as negative control. The plate was covered with a Masterclear real-time PCR film (Eppendorf, Hamburg, Germany) and holes were poked for ventilation. Each experiment contained 10 larvae per immune mutant or background strain, per treatment. Survival experiments were monitored every 8 h for up to 64 h post-infection. Experiments were repeated at least three times.

### 2.5. Statistical Analysis

Statistical tests for gene transcript level analysis were performed using one-way analysis of variance (ANOVA) and a Tukey post-hoc test for multiple comparisons. Statistics for survival experiments were carried out using log-rank (Mantel-Cox) and Chi-square tests. All statistical tests were conducted using the GraphPad Prism 7 software (GraphPad Software, La Jolla, CA, USA).

## 3. Results

### 3.1. Infection with S. carpocapsae Axenic Nematodes Results in Elevated Transcript Levels of Immune-Related Signaling Pathway Genes in D. melanogaster Larvae

**Figure 1:**
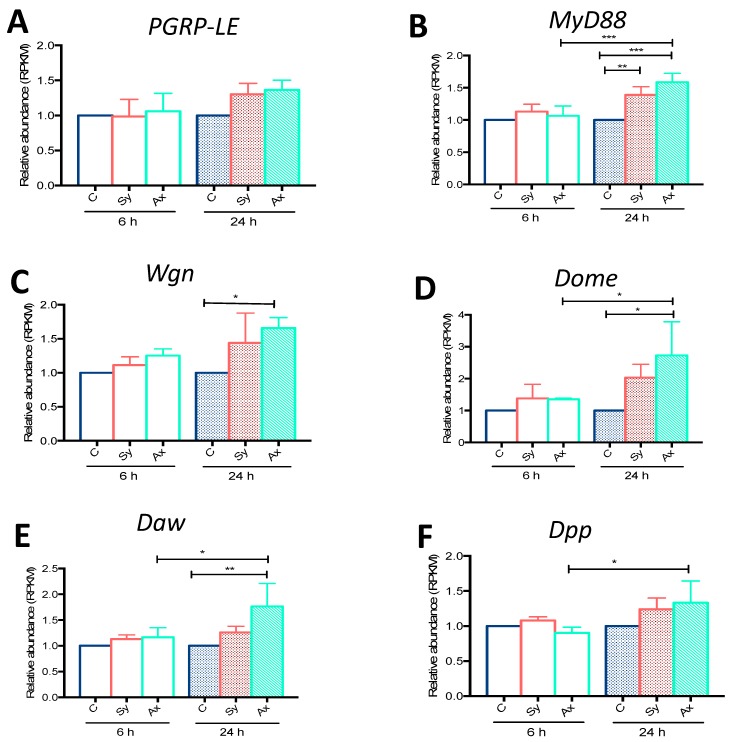
Relative gene transcript levels obtained using RNA-sequencing analyses on *Drosophila melanogaster* larvae infected with *Steinernema carpocapsae* symbiotic or axenic nematodes [7]. Relative transcript (Reads Per Kilobase Million; RPKM) levels of (**A**) *PGRP-LE*; (**B**) *MyD88*; (**C**) *Wengen* (*Wgn*); (**D**) *Domeless* (*Dome*); (**E**) *Dawdle* (*Daw*); and (**F**) *Decapentaplegic* (*Dpp*) were estimated in *D. melanogaster* larvae (Oregon strain) infected with 100 symbiotic (Sy) or axenic (Ax) *S. carpocapsae* nematodes at 6 and 24 h post-infection. Treatment with water served as control (**C**). Relative abundance was estimated as a ratio compared to the uninfected control larvae at 6 and 24 h post-treatment. Values represent means from three biological replicates and error bars represent standard deviations. Data were analyzed using one-way analysis of variance (ANOVA) and a Tukey post-hoc test for multiple comparisons using GraphPad Prism 7 software. * *p* < 0.05, ** *p* < 0.01, *** *p* < 0.001. Non-significant differences (*p* > 0.05) are not shown.

**Figure A1:**
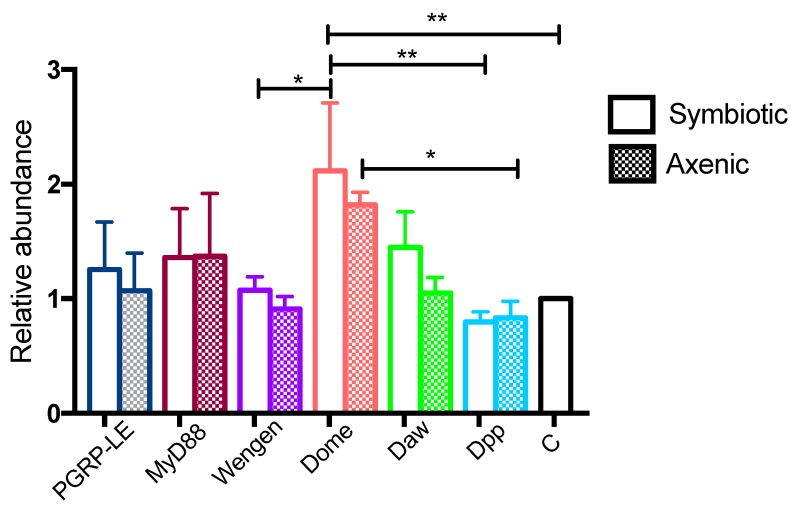
Validation of RNA-sequencing gene transcript levels in *Drosophila melanogaster* larvae infected with *Steinernema carpocapsae* symbiotic or axenic nematodes using qRT-PCR. Relative transcript levels of *PGRP-LE*, *MyD88*, *Wengen*, *Domeless*, *Dawdle*, and *Decapentaplegic* genes in *D. melanogaster* larvae infected with symbiotic or axenic *S. carpocapsae* nematodes at 24 h post-infection. Transcript levels are shown as relative abundance normalized to the housekeeping *RpL32* gene and expressed as a ratio compared to the uninfected control larvae for each strain. Values of uninfected control (C) samples are set to one. Values represent means from four biological replicates and error bars represent standard deviations. Data were analyzed using one-way analysis of variance (ANOVA) and a Tukey post-hoc test for multiple comparisons using GraphPad Prism 7 software. * *p* < 0.05, ** *p* < 0.01, *** *p* < 0.001. Non-significant differences (*p* > 0.05) are not shown.

We plotted the RPKM values for *PGRP-LE* (Imd), *MyD88* (Toll), *Wgn* (Jnk), *Dome* (Jak/Stat), *Daw* (TGFβ, Activin), and *Dpp* (TGFβ, BMP) from *D. melanogaster* Oregon larvae infected for 6 or 24 h with either symbiotic or axenic *S. carpocapsae*. At 6 h post-infection, we found no difference in the expression of any of these genes in larvae infected with symbiotic or axenic nematodes compared to uninfected controls (Figure 1). At 24 h post-infection, the transcript levels of *PGRP-LE* were slightly increased in larvae infected with symbiotic or axenic nematodes compared to the control larvae, although this induction was not statistically significant (*p* = 0.2984 and *p* = 0.1554, respectively; Figure 1A). However, at 24 h post-infection the transcript levels of *MyD88* were higher in larvae challenged with symbiotic (*p* = 0.0087) or axenic (*p* = 0.003) nematodes compared to controls (Figure 1B). Also, the transcript levels of *MyD88* were significantly increased in axenic *S. carpocapsae* infected larvae at 24 h compared to the 6 h time-point (*p* = 0.008; Figure 1B). Transcript levels of *Wgn* were significantly increased at 24 h post-axenic nematode infection compared to uninfected larvae (*p* = 0.0165; Figure 1C). Upon symbiotic nematode infection, *Wgn* was slightly upregulated compared to controls, but this increase was not statistically significant (*p* = 0.1474; Figure 1C). *Dome* and *Daw* were significantly upregulated at 24 h post-axenic nematode infection compared to control larvae (*p* = 0.0108; Figure 1D, *p* = 0.0068; Figure 1E, respectively). *Dome, Daw*, and *Dpp* were upregulated in larvae infected with axenic nematodes at 24 h compared to 6 h (*p* = 0.0474; Figure 1D, *p* = 0.0353; Figure 1E, *p* = 0.0372; Figure 1F). We then validated the induction of all immune-related genes using quantitative RT-PCR and gene-specific primers (Figure A1). Consistent with the RNA-seq data, we did not find any differences in *PGRP-LE* transcript levels between larvae infected with symbiotic or axenic nematodes (Figure A1). In contrast to the RNA-seq, we did not find any differences in *MyD88*, *Wgn*, *Daw*, or *Dpp* transcript levels between uninfected control larvae and those infected with symbiotic nematodes. However, we found significant differences in *Dome* transcript levels in uninfected controls and larvae infected with symbiotic nematodes (*p* = 0.0096; Figure A1). Interestingly, qRT-PCR data showed a significant increase in the transcript levels of *Dome* compared to *Wgn* (*p* = 0.0193) and *Dpp* (*p* = 0.0014) in larvae infected with symbiotic nematodes (Figure A1). In larvae infected with axenic nematodes, *Dome* upregulation was significantly higher than *Dpp* (*p* = 0.0324; Figure A1). These results indicate that infection of *D. melanogaster* larvae with *S. carpocapsae* symbiotic or axenic nematodes results in upregulation of *MyD88*, *Wgn*, *Dome*, *Daw*, and *Dpp* (but not *PGRP-LE*), which are key components of signaling pathways that participate in innate immune processes.

### 3.2. D. melanogaster Imd, Jnk, and TGFβ Pathway Mutants Exhibit Enhanced Survival to Infection by S. carpocapsae Nematodes

**Figure 2:**
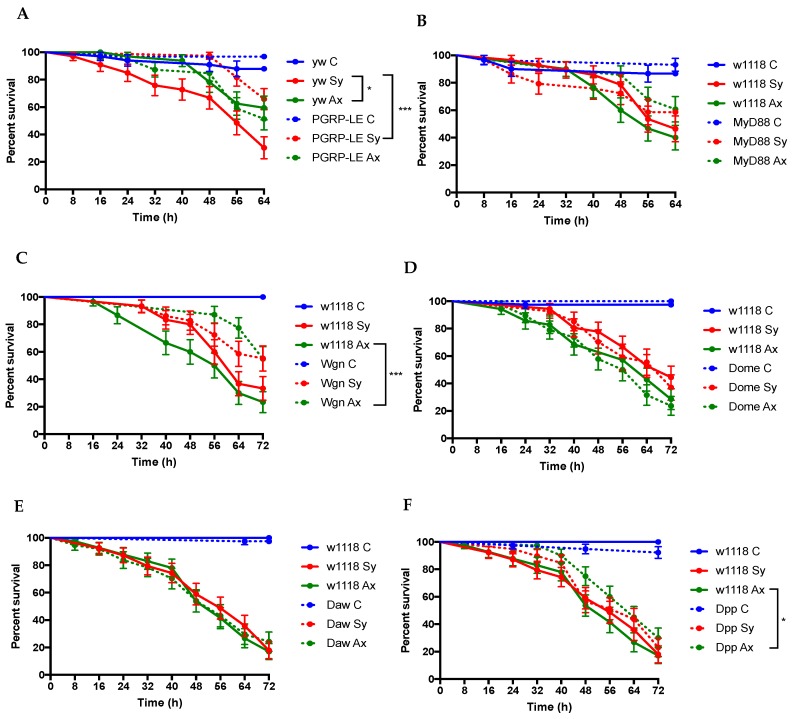
Survival response of *Drosophila melanogaster* immune mutant larvae infected with *Steinernema carpocapsae* symbiotic or axenic nematodes. Survival of late second to early third instar *D. melanogaster* larvae mutant for (**A**) *PGRP-LE*; (**B**) *MyD88*; (**C**) *Wengen* (*Wgn*); (**D**) *Domeless* (*Dome*); (**E**) *Dawdle* (*Daw*); or (**F**) *Decapentaplegic* (*Dpp*) genes, infected with symbiotic (Sy) or axenic (Ax) *S. carpocapsae* nematodes. *Wgn*-RNAi and UAS-*Dome^DN^* were crossed with ubiquitous Actin-Gal4 driver. Treatment with water served as control (**C**). Larval survival was monitored every 8 h for up to 64 h post-infection. Values represent percent survival of infected larvae and data were analyzed using the Log-Rank test using GraphPad Prism 7 software. The means from three independent experiments are shown and bars represent standard errors. * *p* < 0.05, ** *p* < 0.01, *** *p* < 0.001. Non-significant differences (*p* > 0.05) are not shown.

**Figure A2:**
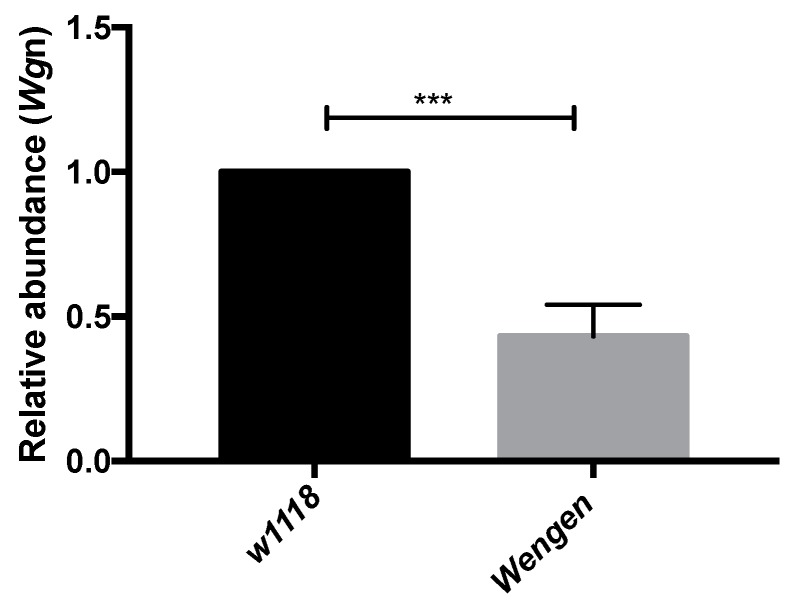
Validation of *Wengen*-RNAi line. Relative transcript levels of *Wengen* (*Wgn*) were measured in larval progeny of *w^1118^* and *Wgn*-RNAi flies crossed with Actin-Gal4 individuals. Transcript levels are represented relative to housekeeping gene *RpL32*, and as a ratio compared to the progeny from the control cross. Values represent means from three replicates and error bar represents standard deviation. Data analysis was performed using unpaired *t*-test using GraphPad Prism 7 software. *** *p* < 0.001.

We infected *D. melanogaster PGRP-LE*, *Myd88*, *Wgn*, *Dome*, *Daw*, and *Dpp* loss-of-function mutant larvae and individuals from their respective background strains with *S. carpocapsae* symbiotic or axenic nematodes and monitored their survival response every 8 h for three days. Upon symbiotic nematode infection, the survival rate for *PGRP-LE* mutant larvae was significantly higher compared to their controls (*p* = 0.0005; Figure 2A). However, no significant differences in survival between *PGRP*-*LE* mutants and their controls were observed upon axenic nematode infection (*p* > 0.05; Figure 2A). In contrast, there was no significant difference between the survival ability of *MyD88* mutant larvae and their background controls following infection with either symbiotic or axenic *S. carpocapsae* nematodes (*p* = 0.607 and *p* = 0.0804, respectively; Figure 2B). After we confirmed significant reduction in *Wgn* transcript levels in larval progeny of *Wgn*-RNAi flies crossed with Actin-Gal4 compared to larval progeny of *w^1118^* flies crossed with this driver line (Figure A2), we found significantly higher survival rates for larvae expressing the *Wgn*-RNAi construct compared to their controls following infection with axenic nematodes (*p* = 0.005; Figure 1C). We further observed no survival differences between *Dome* or *Daw* mutants and their controls in response to symbiotic or axenic nematodes (*p* > 0.05; Figure 2D,E). Although the *Dpp* mutant larvae and their controls succumbed similarly to infection by symbiotic nematodes, these mutants survived better the infection with axenic nematodes than their control individuals (*p* < 0.0291; Figure 1F). These results indicate that inactivation of immune-related genes regulated by Imd, Jnk, and TGFβ pathways promotes the survival ability of *D. melanogaster* larvae to infection by *S. carpocapsae* nematodes.

## 4. Discussion

A recent study investigating the immune response of *D. melanogaster* larvae to entomopathogenic nematodes focused on the induction of antimicrobial peptides (AMPs) as read-outs of the fly humoral immune response [6]. Here, we show that infection of *D. melanogaster* larvae with *S. carpocapsae* nematodes leads to the transcriptional induction of certain genes regulated by the Toll, Jnk, Jak/Stat, and TGFβ immune signaling pathways, but not of the Imd pathway. We also show that inactivation of *PGRP-LE*, which plays a key role in innate immunity by activating the Imd pathway, promotes the survival of *D. melanogaster* against symbiotic *S. carpocapsae*, whereas inactivation of *Wgn* (Jnk pathway) or *Dpp* (TGFβ pathway) genes promotes larval survival in response to axenic nematode infection. 

The transcriptional regulation of immune genes in insects provides an indication for the potential involvement of specific immune signaling pathways in modulating the immune response against microbial invaders. Here, we used RNA-seq to determine the transcriptional induction of genes encoding receptors or signaling components upstream of the receptors in Imd, Toll, Jnk, Jak/Stat, and TGFβ pathways in *D. melanogaster* wild-type larvae infected with either *S. carpocapsae* symbiotic or axenic nematodes. Because RNA-seq is designed to estimate differential expression of a large number of genes, qRT-PCR can be used to validate the expression data [24]. Results from both RNA-seq and qRT-PCR analyses show a similar trend of upregulation for all genes tested. Using RNA-seq, we consistently found upregulation of *Myd88*, *Wgn*, *Dome*, *Daw*, and *Dpp* genes in larvae responding to axenic nematodes compared to symbiotic nematode infections. These results suggest that certain parasitic nematodes can broadly activate immune signaling in *D. melanogaster* larvae even in the absence of their mutualistic bacteria. Interestingly, *S. carpocapsae* nematodes carrying their mutualistic bacteria were recently shown to upregulate antimicrobial peptide coding genes in *D. melanogaster* larvae [6]. Our findings are in agreement with a previous study on *D. melanogaster* adult flies showing upregulation of certain antimicrobial peptide genes in response to *Heterorhabditis bacteriophora* axenic nematodes [25]. Although these immune signaling pathways are activated by both *S. carpocapsae* and *H. bacteriophora*, our current results indicate that *PGRP-LE*, a receptor of the Imd pathway, is not induced in response to *S. carpocapsae* infection. Thus, we speculate that *S. carpocapsae* nematodes can be detected by an unknown receptor in the Imd pathway, which leads to the induction of AMPs, as previously shown [6,7]. Further studies on the molecules involved in the recognition of entomopathogenic nematodes by the *D. melanogaster* immune system will be a future focus of our research.

The lower immune gene expression in response to symbiotic *S. carpocapsae* in the current study suggests that the mutualistic *X. nematophila* bacteria can interfere with the activation of immune signaling. Indeed, previous studies have shown that *X. nematophila* secretes a variety of toxins and virulence factors, many of which act on the insect immune system, thereby suppressing host immune genes that regulate important functions for fighting off the infection [26,27]. For example, the suppression of the *Spodoptera exigua* antimicrobial peptide genes by *X. nematophila* has been attributed to the activity of the metabolite benzylideneacetone, which is produced by the pathogen and acts through the eicosanoid pathway [26]. Also, suppression of *cecropin* expression in *Manduca sexta* is due to the presence of lrp, a global transcription factor in *X. nematophila* [27]. Interestingly, we have found that of the six immune genes tested, only *PGRP-LE* failed to show significant changes in transcript levels in response to *S. carpocapsae* infection. A previous study has demonstrated that the antimicrobial peptide gene *diptericin* is upregulated in *D. melanogaster* wild-type flies in response to *X. nematophila* infection and inactivation of *diptericin* confers high sensitivity to the mutant flies, suggesting that the Imd pathway is not only activated in response to *X. nematophila*, but it can also regulate the fly survival response to this pathogen [28]. The induction of immune genes in Toll, Jnk, Jak/Stat, and TGFβ signaling in our experiments suggests that these pathways could potentially be involved in the *D. melanogaster* response to *S. carpocapsae* nematode infection. 

We further tested whether inactivating any of these genes affects the survival ability of *D. melanogaster* larvae to the nematode parasites. We found delayed mortality of *PGRP-LE* mutants compared to their background controls upon infection with symbiotic, but not axenic, *S. carpocapsae* nematode infection. These results indicate that upon inactivation of Imd signaling, the presence of *X. nematophila* bacteria in *S. carpocapsae* nematodes confers a pathogenicity disadvantage towards *D. melanogaster*. Previously, inactivation of either Toll or Imd pathways had no effect on the survival of *D. melanogaster* larvae upon infection with symbiotic *H. bacteriophora* [29]. These findings in combination with our current results suggest that the two entomopathogenic nematodes, *H. bacteriophora* and *S. carpocapsae*, elicit different responses in *D. melanogaster*, and that Toll and Imd signaling might participate in modulating distinct aspects of the insect anti-nematode immune response depending on the nematode species encountered. The conflicting results showing lack of *PGRP-LE* transcriptional induction by *S. carpocapsae* and the survival advantage of *PGRP*-*LE* deficient mutant larvae to *S. carpocapsae* infection suggests the existence of another Imd component that participates in the interaction with these parasitic nematodes. 

In addition, infection with symbiotic *H. bacteriophora* elicits the transcriptional activation of distinct antimicrobial peptide genes in *D. melanogaster* larvae and adults. For example, *metchnikowin* was induced in *D. melanogaster* larvae in response to symbiotic *H. bacteriophora*, but very low to no induction was observed in adult flies [25,29]. Also, our finding that *Dpp* mutants have increased survival upon infection with axenic *S. carpocapsae* is in disagreement with our recent results showing that inactivation of *Dpp* decreases the survival ability of the mutant flies in response to *H. bacteriophora* axenic nematodes [30]. Thus, the developmental stage of the insect host can also regulate the types and magnitude of immune gene activation and response triggered against entomopathogenic nematode infection. 

Interestingly, we find that inactivation of *Wgn* encoding the sole tumor necrosis factor receptor in *D. melanogaster* promotes the survival of larvae in response to infection by axenic *S. carpocapsae*, suggesting that Jnk signaling, which is involved in several *D. melanogaster* processes such as stress response, morphogenesis, and wound healing [31,32,33], might also interfere with the *D. melanogaster* response to certain nematode parasites. The induction of the Jnk-regulated pathway gene, *puckered*, in *D. melanogaster* adult flies in response to axenic *H. bacteriophora* reinforces the notion that Jnk signaling, through an unknown mechanism, might participate in the interaction with entomopathogenic nematodes [25]. 

Future work will focus on understanding the connection between immune pathway activation in *D. melanogaster* and regulation of the systemic immune function against entomopathogenic nematodes. It will be of particular interest to identify those genes that serve to recognize molecular patterns of entomopathogenic nematodes. The identification and characterization of the immune signaling molecular components that participate in the *D. melanogaster* defense against potent parasitic nematodes will potentially reveal novel anti-nematode mechanisms in other insects and perhaps even mammals.

## 5. Introduction

In this study, we examined the regulation of *PGRP-LE* (Imd pathway), *MyD88* (Toll pathway), *Wgn* (Jnk pathway), *Dome* (Jak/Stat pathway), *Daw* (Activin, TGFβ pathway), and *Dpp* (BMP, TGFβ pathway) genes in *D. melanogaster* larvae responding to infection with *S. carpocapsae* symbiotic or axenic nematodes. We also measured the survival response of mutant larvae against the parasitic nematodes. We have found upregulation of *MyD88*, *Wgn*, *Dome*, *Daw*, and *Dpp*, but not *PGRP-LE*, in *D. melanogaster* infected with the nematode parasites. We have also shown that inactivation of *PGRP-LE*, *Wgn*, and *Dpp* prolongs the survival of nematode-infected larvae. This work demonstrates the modulation of the *D. melanogaster* immune signaling pathways during infection with *S. carpocapsae* entomopathogenic nematodes.

## Appendix B

### Validation of RNA-Seq Data Using qRT-PCR

We collected four *D. melanogaster* larvae per treatment at 24 h post-infection with 100 symbiotic or axenic nematodes. Total RNA was extracted using the Invitrogen^TM^ Ambion Trizol^TM^ reagent following the manufacturer’s instructions. RNA was eluted in 30 µL nuclease-free water and concentration was measured using a NanoDrop (Thermo Scientific, Waltham, MA, USA). Complementary DNA (cDNA) was prepared using the iScript^TM^ cDNA synthesis kit (Bio-Rad, Hercules, CA, USA) and 500 ng of RNA sample in a total reaction volume of 20 µL. cDNA synthesis was carried out on a C1000 Thermal Cycler (Bio-Rad). PCR cycles included 25 °C for 5 min, 46 °C for 20 min, and 95 °C for 1 min. The resulting cDNA samples were diluted 1:10 in nuclease-free water and 1 µL was used as template for qRT-PCR experiments using the iTaq™ Universal SYBR Green Supermix (Bio-Rad) on a CFX96™ Real-Time System (Bio-Rad). The total reaction volume was 20 µL and samples were run in technical duplicates for each set of primers (Table A2).

**Table A1:** *Drosophila melanogaster* strains used in this study.

Gene	Mutant Genotype	Nature of Mutation	Background Strain
*PGRP-LE*	y^1^ w^67c23^ PGRP-LE^112^	P-element activity	*yw*
*MyD88*	NA	P-element activity	*w^1118^*
*Wengen*	P{GD3427}v9152	Transposable element insertion	*w^1118^*
*Domeless*	NA	Homozygous viable	*w^1118^*
*Dawdle*	w^*^; P{GawB}daw^NP4661^/CyO	P-element insertion	*w^1118^*
*Decapentaplegic*	NA	Spontaneous	*w^1118^*

**Table A2:** Primer sequences used in quantitative RT-PCR assays.

Gene	Accession Number	Primer	Sequence	Tm (°C)
*PGRP-LE*	CG8995	Forward Reverse	ATTGCAGAGTCCTCGGTTGTG TTCACTGGTATTTTGGTCGGC	61
*MyD88*	CG2078	Forward Reverse	ATCTGGAACACTTCCTGGGC CCACGAGAGCAGTCTGTCG	61
*Wengen*	CG6531	Forward Reverse	ACCATCTGCGGTTCCATATACG CTGCTCATACTCGGAGGACTT	61
*Domeless*	CG14226	Forward Reverse	GGCGGCGACTTTAATCTGAG GGTGTTGTTCAGGATTCGGAT	61
*Dawdle*	CG16987	Forward Reverse	GGTGGATCAGCAGAAGGACT CCCACTGATCCAGTGTTTGA	61
*Decapentaplegic*	CG9885	Forward Reverse	CCTTGGAGCCTCTGTCGAT TGCACTCTGATCTGGGATTTT	61
*RpL32*	CG7939	Forward Reverse	GATGACCATCCGCCCAGCA CGGACCGACAGCTGCTTGGC	61
